# Ethyl 1-[3-(2-oxopyrrolidin-1-yl)prop­yl]-2-phenyl-1*H*-benzimidazole-5-carboxyl­ate

**DOI:** 10.1107/S1600536811054833

**Published:** 2012-01-07

**Authors:** Yeong Keng Yoon, Mohamed Ashraf Ali, Tan Soo Choon, Safra Izuani Jama Asik, Ibrahim Abdul Razak

**Affiliations:** a Institute for Research in Molecular Medicine, Universiti Sains Malaysia, Minden 11800, Penang, Malaysia; b School of Physics, Universiti Sains Malaysia, 11800 USM, Penang, Malaysia

## Abstract

In the title compound, C_23_H_25_N_3_O_3_, the benzimidazole ring system is essentially planar [maximum deviation = 0.0240 (18) Å]. The mean plane through this ring system forms a dihedral angle of 42.23 (7)° with the benzene ring. The pyrrolidine ring is in an envelope conformation with the flap atom disordered over two sites with occupancies of 0.813 (11) and 0.187 (11). In the crystal, weak C—H⋯O hydrogen bonds form *R*_2_^2^(10) ring motifs, which are connected by further C—H⋯O inter­actions, forming ribbons along the *b* axis. The crystal structure is further stabilized by weak π–π inter­actions involving the imidazole and benzene rings of the benzimidazole ring system [centroid–centroid distances = 3.6788 (11) and 3.6316 (10) Å] and weak C—H⋯π inter­actions.

## Related literature

For the biological activity of benzimidazole derivatives, see: Ozden *et al.* (2008 ▶); Garuti *et al.* (2000 ▶); Rao *et al.* (2002 ▶); Thakurdesai *et al.* (2007 ▶). For ring conformations, see: Cremer & Pople (1975 ▶). For the stability of the temperature controller used in the data collection, see: Cosier & Glazer (1986 ▶). For hydrogen-bond motifs, see: Bernstein *et al.* (1995 ▶).
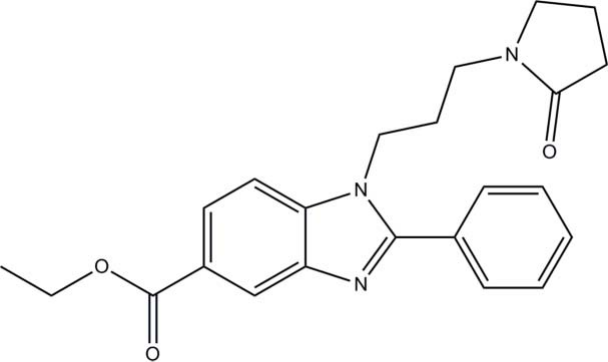


## Experimental

### 

#### Crystal data

C_23_H_25_N_3_O_3_
*M* *_r_* = 391.46
Triclinic, 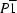

*a* = 9.9469 (3) Å
*b* = 10.5845 (3) Å
*c* = 11.3184 (3) Å
α = 69.679 (1)°
β = 67.374 (1)°
γ = 70.135 (1)°
*V* = 1001.77 (5) Å^3^
*Z* = 2
Mo *K*α radiation
μ = 0.09 mm^−1^
*T* = 100 K
0.39 × 0.36 × 0.25 mm

#### Data collection

Bruker SMART APEXII CCD area-detector diffractometer
Absorption correction: multi-scan (*SADABS*; Bruker, 2009 ▶) *T*_min_ = 0.967, *T*_max_ = 0.979
17976 measured reflections
4530 independent reflections
3768 reflections with *I* > 2σ(*I*)
*R*_int_ = 0.038

#### Refinement

*R*[*F* ^2^ > 2σ(*F* ^2^)] = 0.055
*wR*(*F* ^2^) = 0.148
*S* = 1.04
4530 reflections
273 parameters
H-atom parameters constrained
Δρ_max_ = 0.48 e Å^−3^
Δρ_min_ = −0.29 e Å^−3^

### 

Data collection: *APEX2* (Bruker, 2009 ▶); cell refinement: *SAINT* (Bruker, 2009 ▶); data reduction: *SAINT*; program(s) used to solve structure: *SHELXTL* (Sheldrick, 2008 ▶); program(s) used to refine structure: *SHELXTL*; molecular graphics: *SHELXTL*; software used to prepare material for publication: *SHELXTL* and *PLATON* (Spek, 2009 ▶).

## Supplementary Material

Crystal structure: contains datablock(s) global, I. DOI: 10.1107/S1600536811054833/lh5398sup1.cif

Structure factors: contains datablock(s) I. DOI: 10.1107/S1600536811054833/lh5398Isup2.hkl

Supplementary material file. DOI: 10.1107/S1600536811054833/lh5398Isup3.cml

Additional supplementary materials:  crystallographic information; 3D view; checkCIF report

## Figures and Tables

**Table 1:** Hydrogen-bond geometry (Å, °) *Cg* is the centroid of the C1–C6 ring.

*D*—H⋯*A*	*D*—H	H⋯*A*	*D*⋯*A*	*D*—H⋯*A*
C5—H5*A*⋯O3^i^	0.95	2.32	3.266 (2)	172
C16—H16*B*⋯O3^ii^	0.98	2.56	3.341 (3)	137
C19—H19*B*⋯O3^i^	0.99	2.57	3.391 (3)	141
C10—H10*A*⋯*Cg*^iii^	0.95	2.90	3.516 (2)	124

Symmetry codes: (i) 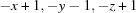; (ii) 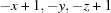; (iii) 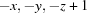.

**Fig. 1.**
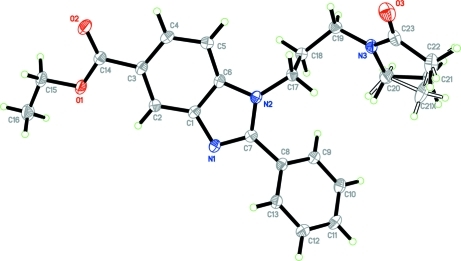
The molecular structure of the title compound, showing 50% probability displacement ellipsoids. Hydrogen atoms are shown as spheres of arbitrary radius. The disorder is shown with open bonds.

**Fig. 2.**
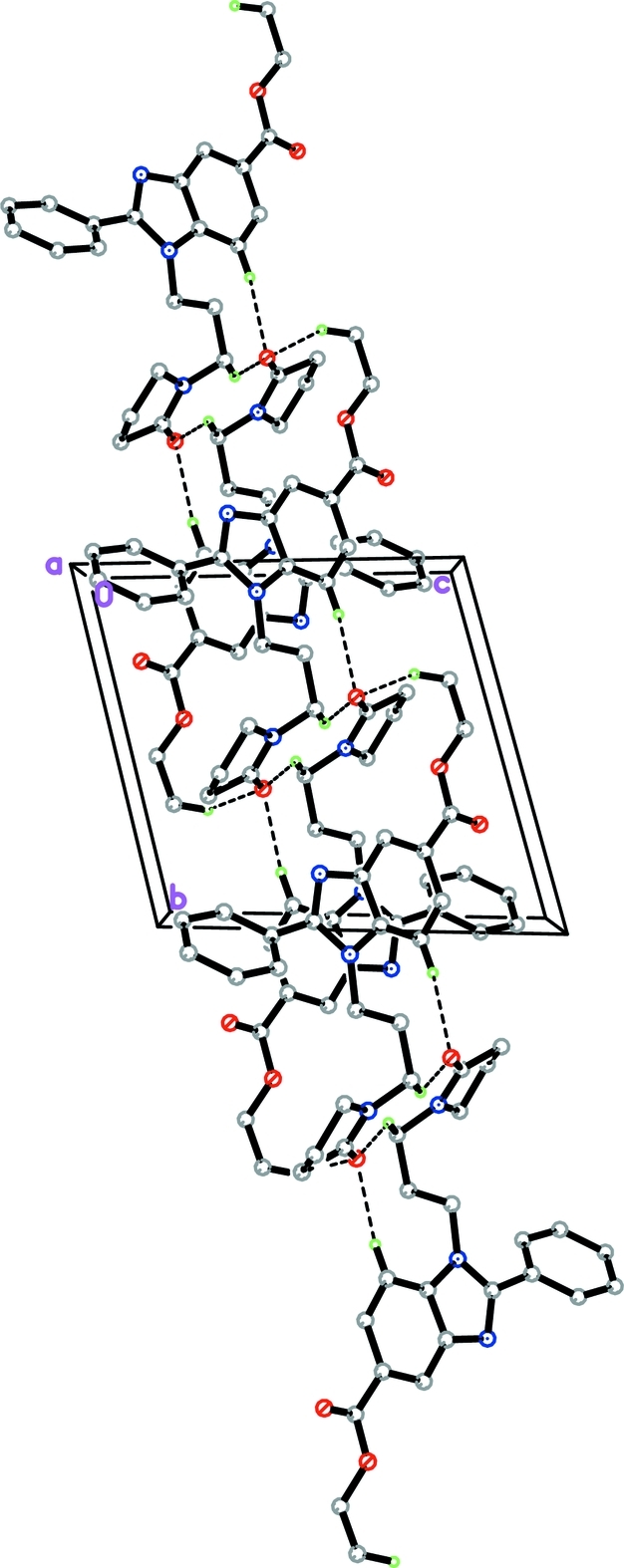
The crystal packing, viewed along the a-axis, showing the molecules are connected into ribbon along b axis. Hydrogen bonds are shown as dashed lines.

## supplementary crystallographic information

### Comment

Benzimidazoles are a class of bioactive heterocyclic compounds which exhibit a wide range of activities such as antibacterial (Ozden *et al.*, 2008), antiproliferatives (Garuti *et al.*, 2000), anti-HIV (Rao *et al.*, 2002) and anti-inflammatory (Thakurdesai *et al.*, 2007). In view of their importance, the crystal structure determination of the title compound was carried out and the results are presented here.

In the title molecule, Fig. 1, the benzimidazole, (N1–N2/C1–C7) ring is essentially planar with maximum deviation of 0.0240 (18) Å for atom C5. The mean plane through this ring makes a dihedral angle of 42.23 (7)° with the benzene, (C8–C13) ring. Atom C21 is disordered (Fig. 1) over two positions, with occupancy ratios of 0.813 (11):0.187 (11). The disordered pyrrolidine ring adopts an envelope conformation with puckering parameters Q = 0.2836 Å, φ = 249.7429° with C21 at the flap and Q = 0.2163 Å, φ = 77.1714° with C21X at the flap (Cremer & Pople, 1975).

In the crystal packing (Fig. 2), R^2^_2_(10) ring motifs (Bernstein *et al.*, 1995) are formed by C19—H19B···O3(1-x,-1-y,1-z) intermolecular interactions. C5—H5A···O3(1-x,-1-y,1-z) and C16—H16B···O3 (1-x,-y,1-z) interactions further link the molecules into ribbon along the *b* axis. π–π stacking interactions are observed within the benzimidazole ring system between the imadazole (N1–N2/C1/C6–C7); centroid *Cg*1) and the benzene (C1–C6; centroid *Cg*2) rings with a *Cg*1···*Cg*2 (1-x,-y,1-z) distance of 3.6788 (11) and between the benzene rings with a *Cg*2···*Cg*2(1-x,-y,1-z) distance of 3.6316 (10). The crystal packing is further stabilized by weak C—H···π interactions (Table 1).

### Experimental

Ethyl 3-amino-4-(3(2-oxopyrrolidin-1yl)propylamino)benzoate (0.84 mmol) and sodium metabisulfite adduct of benzaldehyde (1.68 mmol) were dissolved in DMF. The reaction mixture was reflux at 130 °C for 2 hrs. After completion, the reaction mixture was diluted in Ethyl acetate (20 mL) and washed with water (20 mL). The organic layer was collected, dried over Na_2_SO_4_ and the evaporated in vacuo to yield the product. The product was recrystallised from Ethyl acetate.

### Refinement

All H atoms positioned geometrically and refined using a riding model with with C–H = 0.95–0.99 Å. The *U*_iso_ values were constrained to be 1.5*U*_eq_ (methyl-H atom) and 1.2*U*_eq_ (other H atoms). The rotating model group was applied for the methyl group.

### Crystal data

**Table:**

C_23_H_25_N_3_O_3_	*Z* = 2
*M**_r_* = 391.46	*F*(000) = 416
Triclinic, *P*1	*D*_x_ = 1.298 Mg m^−^^3^
Hall symbol: -P 1	Mo *K*α radiation, λ = 0.71073 Å
*a* = 9.9469 (3) Å	Cell parameters from 8488 reflections
*b* = 10.5845 (3) Å	θ = 2.5–30.0°
*c* = 11.3184 (3) Å	µ = 0.09 mm^−^^1^
α = 69.679 (1)°	*T* = 100 K
β = 67.374 (1)°	Block, yellow
γ = 70.135 (1)°	0.39 × 0.36 × 0.25 mm
*V* = 1001.77 (5) Å^3^	

### Data collection

**Table:**

Bruker SMART APEXII CCD area-detector diffractometer	4530 independent reflections
Radiation source: fine-focus sealed tube	3768 reflections with *I* > 2σ(*I*)
graphite	*R*_int_ = 0.038
φ and ω scans	θ_max_ = 27.5°, θ_min_ = 2.0°
Absorption correction: multi-scan (*SADABS*; Bruker, 2009)	*h* = −12→12
*T*_min_ = 0.967, *T*_max_ = 0.979	*k* = −13→13
17976 measured reflections	*l* = −14→14

### Refinement

**Table:**

Refinement on *F*^2^	Primary atom site location: structure-invariant direct methods
Least-squares matrix: full	Secondary atom site location: difference Fourier map
*R*[*F*^2^ > 2σ(*F*^2^)] = 0.055	Hydrogen site location: inferred from neighbouring sites
*wR*(*F*^2^) = 0.148	H-atom parameters constrained
*S* = 1.04	*w* = 1/[σ^2^(*F*_o_^2^) + (0.0797*P*)^2^ + 0.518*P*] where *P* = (*F*_o_^2^ + 2*F*_c_^2^)/3
4530 reflections	(Δ/σ)_max_ < 0.001
273 parameters	Δρ_max_ = 0.48 e Å^−^^3^
0 restraints	Δρ_min_ = −0.29 e Å^−^^3^

### Special details

**Table:**

Experimental. The crystal was placed in the cold stream of an Oxford Cryosystems Cobra open-flow nitrogen cryostat (Cosier & Glazer, 1986) operating at 100.0 (1) K.
Geometry. All esds (except the esd in the dihedral angle between two l.s. planes) are estimated using the full covariance matrix. The cell esds are taken into account individually in the estimation of esds in distances, angles and torsion angles; correlations between esds in cell parameters are only used when they are defined by crystal symmetry. An approximate (isotropic) treatment of cell esds is used for estimating esds involving l.s. planes.
Refinement. Refinement of F^2^ against ALL reflections. The weighted R-factor wR and goodness of fit S are based on F^2^, conventional R-factors R are based on F, with F set to zero for negative F^2^. The threshold expression of F^2^ > 2sigma(F^2^) is used only for calculating R-factors(gt) etc. and is not relevant to the choice of reflections for refinement. R-factors based on F^2^ are statistically about twice as large as those based on F, and R- factors based on ALL data will be even larger.

### Fractional atomic coordinates and isotropic or equivalent isotropic displacement parameters (Å^2^)

**Table:**

	*x*	*y*	*z*	*U*_iso_*/*U*_eq_	Occ. (<1)
O1	0.51811 (13)	0.43185 (12)	0.18372 (11)	0.0238 (3)	
O2	0.72338 (14)	0.26966 (13)	0.11427 (12)	0.0278 (3)	
O3	0.38369 (15)	−0.62929 (14)	0.65226 (14)	0.0336 (3)	
N1	0.16338 (15)	0.14323 (14)	0.56013 (13)	0.0183 (3)	
N2	0.25147 (15)	−0.07610 (14)	0.53712 (13)	0.0173 (3)	
N3	0.17813 (16)	−0.48401 (14)	0.59087 (14)	0.0223 (3)	
C1	0.29539 (17)	0.13446 (17)	0.45592 (15)	0.0175 (3)	
C2	0.37149 (18)	0.23727 (17)	0.37207 (15)	0.0180 (3)	
H2A	0.3329	0.3305	0.3794	0.022*	
C3	0.50599 (18)	0.19825 (17)	0.27734 (15)	0.0190 (3)	
C4	0.56275 (18)	0.05964 (17)	0.26704 (15)	0.0197 (3)	
H4A	0.6561	0.0359	0.2028	0.024*	
C5	0.48733 (18)	−0.04269 (17)	0.34713 (15)	0.0192 (3)	
H5A	0.5251	−0.1356	0.3388	0.023*	
C6	0.35264 (18)	−0.00195 (17)	0.44091 (15)	0.0173 (3)	
C7	0.14155 (17)	0.01636 (17)	0.60518 (15)	0.0178 (3)	
C8	0.01066 (17)	−0.01765 (16)	0.71822 (15)	0.0182 (3)	
C9	−0.07626 (18)	−0.09810 (17)	0.71996 (16)	0.0201 (3)	
H9A	−0.0472	−0.1401	0.6493	0.024*	
C10	−0.20564 (19)	−0.11690 (18)	0.82509 (16)	0.0235 (4)	
H10A	−0.2640	−0.1723	0.8262	0.028*	
C11	−0.24936 (19)	−0.05501 (19)	0.92789 (17)	0.0254 (4)	
H11A	−0.3375	−0.0681	0.9995	0.030*	
C12	−0.1640 (2)	0.02624 (19)	0.92607 (17)	0.0251 (4)	
H12A	−0.1943	0.0696	0.9960	0.030*	
C13	−0.03462 (19)	0.04401 (17)	0.82207 (16)	0.0212 (3)	
H13A	0.0238	0.0990	0.8217	0.025*	
C14	0.59541 (18)	0.30010 (17)	0.18400 (15)	0.0198 (3)	
C15	0.5951 (2)	0.53843 (19)	0.08991 (17)	0.0267 (4)	
H15A	0.6270	0.5271	−0.0009	0.032*	
H15B	0.6852	0.5317	0.1115	0.032*	
C16	0.4870 (2)	0.6758 (2)	0.09972 (18)	0.0315 (4)	
H16A	0.5293	0.7494	0.0285	0.047*	
H16B	0.4686	0.6921	0.1853	0.047*	
H16C	0.3923	0.6758	0.0918	0.047*	
C17	0.26948 (18)	−0.22474 (16)	0.55756 (15)	0.0179 (3)	
H17A	0.2068	−0.2620	0.6475	0.021*	
H17B	0.3754	−0.2737	0.5509	0.021*	
C18	0.22422 (18)	−0.25223 (16)	0.45568 (15)	0.0189 (3)	
H18A	0.1154	−0.2121	0.4693	0.023*	
H18B	0.2782	−0.2050	0.3659	0.023*	
C19	0.25864 (19)	−0.40646 (17)	0.46449 (16)	0.0212 (3)	
H19A	0.2324	−0.4175	0.3933	0.025*	
H19B	0.3677	−0.4461	0.4495	0.025*	
C20	0.0140 (2)	−0.45478 (19)	0.64291 (19)	0.0285 (4)	
H20A	−0.0309	−0.4359	0.5724	0.034*	0.813 (11)
H20B	−0.0264	−0.3741	0.6821	0.034*	0.813 (11)
H20C	−0.0269	−0.4725	0.5877	0.034*	0.187 (11)
H20D	−0.0269	−0.3596	0.6470	0.034*	0.187 (11)
C21	−0.0168 (3)	−0.5853 (3)	0.7469 (3)	0.0277 (8)	0.813 (11)
H21A	−0.0297	−0.6506	0.7094	0.033*	0.813 (11)
H21B	−0.1075	−0.5643	0.8212	0.033*	0.813 (11)
C21X	−0.0012 (16)	−0.5530 (18)	0.7962 (18)	0.049 (5)	0.187 (11)
H21C	−0.0217	−0.4957	0.8569	0.059*	0.187 (11)
H21D	−0.0842	−0.5996	0.8275	0.059*	0.187 (11)
C22	0.1275 (2)	−0.6456 (2)	0.7924 (2)	0.0345 (5)	
H22A	0.1139	−0.6136	0.8696	0.041*	0.813 (11)
H22B	0.1533	−0.7483	0.8158	0.041*	0.813 (11)
H22C	0.1333	−0.7385	0.7937	0.041*	0.187 (11)
H22D	0.1447	−0.6489	0.8714	0.041*	0.187 (11)
C23	0.2473 (2)	−0.58904 (18)	0.67283 (18)	0.0257 (4)	

### Atomic displacement parameters (Å^2^)

**Table:**

	*U*^11^	*U*^22^	*U*^33^	*U*^12^	*U*^13^	*U*^23^
O1	0.0232 (6)	0.0232 (6)	0.0213 (6)	−0.0115 (5)	−0.0019 (5)	−0.0008 (5)
O2	0.0200 (6)	0.0320 (7)	0.0274 (6)	−0.0106 (5)	−0.0004 (5)	−0.0060 (5)
O3	0.0287 (7)	0.0256 (7)	0.0471 (8)	−0.0023 (6)	−0.0203 (6)	−0.0037 (6)
N1	0.0162 (7)	0.0205 (7)	0.0176 (6)	−0.0064 (5)	−0.0046 (5)	−0.0026 (5)
N2	0.0160 (7)	0.0189 (7)	0.0168 (6)	−0.0057 (5)	−0.0051 (5)	−0.0028 (5)
N3	0.0185 (7)	0.0178 (7)	0.0285 (7)	−0.0053 (5)	−0.0064 (6)	−0.0033 (6)
C1	0.0144 (7)	0.0225 (8)	0.0164 (7)	−0.0051 (6)	−0.0060 (6)	−0.0038 (6)
C2	0.0176 (8)	0.0186 (8)	0.0190 (7)	−0.0046 (6)	−0.0072 (6)	−0.0041 (6)
C3	0.0174 (8)	0.0245 (8)	0.0166 (7)	−0.0082 (6)	−0.0064 (6)	−0.0022 (6)
C4	0.0150 (8)	0.0261 (9)	0.0166 (7)	−0.0051 (6)	−0.0034 (6)	−0.0049 (6)
C5	0.0195 (8)	0.0196 (8)	0.0187 (7)	−0.0031 (6)	−0.0068 (6)	−0.0054 (6)
C6	0.0164 (7)	0.0202 (8)	0.0167 (7)	−0.0069 (6)	−0.0071 (6)	−0.0014 (6)
C7	0.0158 (8)	0.0219 (8)	0.0173 (7)	−0.0043 (6)	−0.0069 (6)	−0.0048 (6)
C8	0.0163 (8)	0.0178 (8)	0.0176 (7)	−0.0036 (6)	−0.0058 (6)	−0.0006 (6)
C9	0.0192 (8)	0.0219 (8)	0.0188 (7)	−0.0061 (6)	−0.0057 (6)	−0.0037 (6)
C10	0.0211 (8)	0.0261 (9)	0.0241 (8)	−0.0110 (7)	−0.0069 (7)	−0.0017 (7)
C11	0.0198 (8)	0.0309 (9)	0.0204 (8)	−0.0097 (7)	−0.0006 (6)	−0.0031 (7)
C12	0.0266 (9)	0.0277 (9)	0.0193 (8)	−0.0085 (7)	−0.0025 (7)	−0.0068 (7)
C13	0.0218 (8)	0.0222 (8)	0.0201 (8)	−0.0090 (7)	−0.0055 (6)	−0.0032 (6)
C14	0.0192 (8)	0.0255 (8)	0.0172 (7)	−0.0084 (7)	−0.0059 (6)	−0.0046 (6)
C15	0.0285 (9)	0.0292 (9)	0.0224 (8)	−0.0171 (8)	−0.0031 (7)	−0.0014 (7)
C16	0.0375 (11)	0.0290 (10)	0.0270 (9)	−0.0158 (8)	−0.0071 (8)	−0.0012 (7)
C17	0.0182 (8)	0.0158 (8)	0.0193 (7)	−0.0046 (6)	−0.0065 (6)	−0.0024 (6)
C18	0.0190 (8)	0.0191 (8)	0.0190 (7)	−0.0051 (6)	−0.0069 (6)	−0.0034 (6)
C19	0.0191 (8)	0.0222 (8)	0.0226 (8)	−0.0050 (7)	−0.0051 (6)	−0.0071 (6)
C20	0.0213 (9)	0.0289 (10)	0.0341 (10)	−0.0085 (7)	−0.0057 (7)	−0.0072 (8)
C21	0.0253 (12)	0.0266 (13)	0.0305 (14)	−0.0117 (9)	−0.0087 (10)	−0.0007 (10)
C21X	0.043 (8)	0.059 (9)	0.039 (8)	−0.036 (7)	−0.001 (6)	0.005 (7)
C22	0.0406 (12)	0.0322 (10)	0.0328 (10)	−0.0193 (9)	−0.0144 (9)	0.0034 (8)
C23	0.0303 (10)	0.0175 (8)	0.0326 (9)	−0.0069 (7)	−0.0136 (8)	−0.0044 (7)

### Geometric parameters (Å, °)

**Table:**

O1—C14	1.344 (2)	C13—H13A	0.9500
O1—C15	1.455 (2)	C15—C16	1.497 (3)
O2—C14	1.213 (2)	C15—H15A	0.9900
O3—C23	1.229 (2)	C15—H15B	0.9900
N1—C7	1.322 (2)	C16—H16A	0.9800
N1—C1	1.388 (2)	C16—H16B	0.9800
N2—C7	1.377 (2)	C16—H16C	0.9800
N2—C6	1.384 (2)	C17—C18	1.529 (2)
N2—C17	1.466 (2)	C17—H17A	0.9900
N3—C23	1.351 (2)	C17—H17B	0.9900
N3—C19	1.453 (2)	C18—C19	1.525 (2)
N3—C20	1.466 (2)	C18—H18A	0.9900
C1—C2	1.396 (2)	C18—H18B	0.9900
C1—C6	1.405 (2)	C19—H19A	0.9900
C2—C3	1.392 (2)	C19—H19B	0.9900
C2—H2A	0.9500	C20—C21	1.507 (3)
C3—C4	1.411 (2)	C20—C21X	1.663 (16)
C3—C14	1.486 (2)	C20—H20A	0.9900
C4—C5	1.380 (2)	C20—H20B	0.9900
C4—H4A	0.9500	C20—H20C	0.9600
C5—C6	1.392 (2)	C20—H20D	0.9600
C5—H5A	0.9500	C21—C22	1.566 (4)
C7—C8	1.476 (2)	C21—H21A	0.9900
C8—C13	1.391 (2)	C21—H21B	0.9900
C8—C9	1.396 (2)	C21X—C22	1.318 (15)
C9—C10	1.394 (2)	C21X—H21C	0.9900
C9—H9A	0.9500	C21X—H21D	0.9900
C10—C11	1.385 (2)	C22—C23	1.514 (3)
C10—H10A	0.9500	C22—H22A	0.9900
C11—C12	1.390 (3)	C22—H22B	0.9900
C11—H11A	0.9500	C22—H22C	0.9601
C12—C13	1.386 (2)	C22—H22D	0.9599
C12—H12A	0.9500		
			
C14—O1—C15	115.89 (13)	C18—C17—H17B	109.3
C7—N1—C1	104.67 (13)	H17A—C17—H17B	108.0
C7—N2—C6	106.22 (13)	C19—C18—C17	112.60 (13)
C7—N2—C17	130.10 (13)	C19—C18—H18A	109.1
C6—N2—C17	123.66 (13)	C17—C18—H18A	109.1
C23—N3—C19	123.22 (15)	C19—C18—H18B	109.1
C23—N3—C20	113.37 (15)	C17—C18—H18B	109.1
C19—N3—C20	123.40 (14)	H18A—C18—H18B	107.8
N1—C1—C2	129.67 (15)	N3—C19—C18	113.27 (13)
N1—C1—C6	110.17 (14)	N3—C19—H19A	108.9
C2—C1—C6	120.16 (14)	C18—C19—H19A	108.9
C3—C2—C1	117.58 (15)	N3—C19—H19B	108.9
C3—C2—H2A	121.2	C18—C19—H19B	108.9
C1—C2—H2A	121.2	H19A—C19—H19B	107.7
C2—C3—C4	120.96 (14)	N3—C20—C21	104.03 (16)
C2—C3—C14	121.55 (15)	N3—C20—C21X	98.5 (5)
C4—C3—C14	117.49 (14)	N3—C20—H20A	110.9
C5—C4—C3	122.24 (15)	C21—C20—H20A	110.9
C5—C4—H4A	118.9	C21X—C20—H20A	137.8
C3—C4—H4A	118.9	N3—C20—H20B	110.9
C4—C5—C6	116.13 (15)	C21—C20—H20B	110.9
C4—C5—H5A	121.9	C21X—C20—H20B	86.5
C6—C5—H5A	121.9	H20A—C20—H20B	109.0
N2—C6—C5	131.52 (15)	N3—C20—H20C	110.6
N2—C6—C1	105.56 (13)	C21—C20—H20C	91.1
C5—C6—C1	122.89 (14)	C21X—C20—H20C	119.9
N1—C7—N2	113.37 (14)	H20B—C20—H20C	125.6
N1—C7—C8	121.39 (14)	N3—C20—H20D	110.5
N2—C7—C8	125.23 (14)	C21—C20—H20D	129.9
C13—C8—C9	119.02 (15)	C21X—C20—H20D	108.2
C13—C8—C7	117.69 (14)	H20A—C20—H20D	89.5
C9—C8—C7	123.04 (14)	H20C—C20—H20D	108.7
C10—C9—C8	120.16 (15)	C20—C21—C22	102.78 (19)
C10—C9—H9A	119.9	C20—C21—H21A	111.2
C8—C9—H9A	119.9	C22—C21—H21A	111.2
C11—C10—C9	120.22 (16)	C20—C21—H21B	111.2
C11—C10—H10A	119.9	C22—C21—H21B	111.2
C9—C10—H10A	119.9	H21A—C21—H21B	109.1
C10—C11—C12	119.86 (15)	C22—C21X—C20	106.7 (10)
C10—C11—H11A	120.1	C22—C21X—H21C	110.4
C12—C11—H11A	120.1	C20—C21X—H21C	110.4
C13—C12—C11	119.93 (16)	C22—C21X—H21D	110.4
C13—C12—H12A	120.0	C20—C21X—H21D	110.4
C11—C12—H12A	120.0	H21C—C21X—H21D	108.6
C12—C13—C8	120.81 (15)	C21X—C22—C23	108.3 (5)
C12—C13—H13A	119.6	C23—C22—C21	103.66 (16)
C8—C13—H13A	119.6	C21X—C22—H22A	80.3
O2—C14—O1	123.01 (15)	C23—C22—H22A	111.0
O2—C14—C3	124.58 (16)	C21—C22—H22A	111.0
O1—C14—C3	112.41 (14)	C21X—C22—H22B	132.2
O1—C15—C16	107.21 (14)	C23—C22—H22B	111.0
O1—C15—H15A	110.3	C21—C22—H22B	111.0
C16—C15—H15A	110.3	H22A—C22—H22B	109.0
O1—C15—H15B	110.3	C21X—C22—H22C	118.6
C16—C15—H15B	110.3	C23—C22—H22C	109.4
H15A—C15—H15B	108.5	C21—C22—H22C	92.5
C15—C16—H16A	109.5	H22A—C22—H22C	125.7
C15—C16—H16B	109.5	C21X—C22—H22D	102.6
H16A—C16—H16B	109.5	C23—C22—H22D	109.4
C15—C16—H16C	109.5	C21—C22—H22D	131.5
H16A—C16—H16C	109.5	H22B—C22—H22D	89.0
H16B—C16—H16C	109.5	H22C—C22—H22D	108.1
N2—C17—C18	111.47 (13)	O3—C23—N3	125.09 (17)
N2—C17—H17A	109.3	O3—C23—C22	126.97 (17)
C18—C17—H17A	109.3	N3—C23—C22	107.94 (15)
N2—C17—H17B	109.3		
			
C7—N1—C1—C2	179.81 (16)	C9—C8—C13—C12	0.0 (2)
C7—N1—C1—C6	−0.59 (17)	C7—C8—C13—C12	174.46 (15)
N1—C1—C2—C3	177.87 (15)	C15—O1—C14—O2	2.4 (2)
C6—C1—C2—C3	−1.7 (2)	C15—O1—C14—C3	−176.97 (13)
C1—C2—C3—C4	0.0 (2)	C2—C3—C14—O2	167.58 (15)
C1—C2—C3—C14	−179.74 (14)	C4—C3—C14—O2	−12.1 (2)
C2—C3—C4—C5	1.5 (2)	C2—C3—C14—O1	−13.1 (2)
C14—C3—C4—C5	−178.76 (14)	C4—C3—C14—O1	167.23 (13)
C3—C4—C5—C6	−1.1 (2)	C14—O1—C15—C16	176.49 (14)
C7—N2—C6—C5	177.41 (16)	C7—N2—C17—C18	103.28 (18)
C17—N2—C6—C5	−1.0 (3)	C6—N2—C17—C18	−78.68 (18)
C7—N2—C6—C1	−0.75 (16)	N2—C17—C18—C19	173.40 (12)
C17—N2—C6—C1	−179.19 (13)	C23—N3—C19—C18	−119.11 (17)
C4—C5—C6—N2	−178.55 (15)	C20—N3—C19—C18	60.0 (2)
C4—C5—C6—C1	−0.7 (2)	C17—C18—C19—N3	61.78 (18)
N1—C1—C6—N2	0.85 (17)	C23—N3—C20—C21	−19.5 (2)
C2—C1—C6—N2	−179.51 (13)	C19—N3—C20—C21	161.3 (2)
N1—C1—C6—C5	−177.51 (14)	C23—N3—C20—C21X	10.4 (9)
C2—C1—C6—C5	2.1 (2)	C19—N3—C20—C21X	−168.8 (9)
C1—N1—C7—N2	0.10 (17)	N3—C20—C21—C22	27.4 (3)
C1—N1—C7—C8	179.80 (13)	C21X—C20—C21—C22	−55.1 (10)
C6—N2—C7—N1	0.42 (18)	N3—C20—C21X—C22	−20.9 (15)
C17—N2—C7—N1	178.73 (14)	C21—C20—C21X—C22	82.6 (17)
C6—N2—C7—C8	−179.26 (14)	C20—C21X—C22—C23	23.0 (16)
C17—N2—C7—C8	−1.0 (3)	C20—C21X—C22—C21	−63.6 (13)
N1—C7—C8—C13	−38.6 (2)	C20—C21—C22—C21X	76.2 (10)
N2—C7—C8—C13	141.03 (16)	C20—C21—C22—C23	−26.5 (3)
N1—C7—C8—C9	135.58 (16)	C19—N3—C23—O3	1.5 (3)
N2—C7—C8—C9	−44.8 (2)	C20—N3—C23—O3	−177.71 (17)
C13—C8—C9—C10	−0.6 (2)	C19—N3—C23—C22	−178.94 (15)
C7—C8—C9—C10	−174.69 (15)	C20—N3—C23—C22	1.9 (2)
C8—C9—C10—C11	0.5 (3)	C21X—C22—C23—O3	162.4 (12)
C9—C10—C11—C12	0.1 (3)	C21—C22—C23—O3	−164.6 (2)
C10—C11—C12—C13	−0.6 (3)	C21X—C22—C23—N3	−17.2 (12)
C11—C12—C13—C8	0.6 (3)	C21—C22—C23—N3	15.8 (2)

### Hydrogen-bond geometry (Å, °)

**Table:**

Cg is the centroid of the C1–C6 ring.

**Table:**

*D*—H···*A*	*D*—H	H···*A*	*D*···*A*	*D*—H···*A*
C5—H5A···O3^i^	0.95	2.32	3.266 (2)	172
C16—H16B···O3^ii^	0.98	2.56	3.341 (3)	137
C19—H19B···O3^i^	0.99	2.57	3.391 (3)	141
C10—H10A···Cg^iii^	0.95	2.90	3.516 (2)	124

Symmetry codes: (i) −*x*+1, −*y*−1, −*z*+1; (ii) −*x*+1, −*y*, −*z*+1; (iii) −*x*, −*y*, −*z*+1.
